# Supplementary material for: Integrated bioinformatics analysis of As, Au, Cd, Pb and Cu heavy metal responsive marker genes through Arabidopsis thaliana GEO datasets
Source: PeerJ. 2019 Mar 18;7:e6495. doi: 10.7717/peerj.6495 (PMC6428040; doi:10.7717/peerj.6495)
Supplement: Table S2 [file peerj-07-6495-s002.doc]

**Table S2. The list of RT-qPCR primers of candidate genes in *A. thaliana*.**

| Gene name | Gene model | Forward Primer | Reward Primer |
| --- | --- | --- | --- |
| NILR1 | AT1G74360 | AAGCTTCAGAGGGAAGGCAC | CTCCCGCCTCCCATGTATTC |
| PGPS1 | AT1G19020 | TGACCAGTGGGACAATAGCG | GGTCGTGTTTGACGTTGCTC |
| WRKY33 | AT2G38470 | ACCATCGGTTGTCCAGTGAG | CGAATCCTGTGGTGCTCTGT |
| BCS1 | AT3G50930 | CTATGGTCAAGCTGCGGTGA | AGGCGGTGCTCTTTGATCTC |
| AR781 | AT2G26530 | CCGCAAAGCTTTTTCCCCAAG | GTCTCCCTCTCCCTCTTTCCT |
| NR1 | AT1G77760 | TCCAGAGGACGAAACCGAGA | ACTCCAACCTTCCTTTGCGA |
| CYP81D8 | AT4G37370 | GACCGACACATCAGCGGTTA | ATGGGAGCCGCAGGATAAAG |
| EAP1 | AT3G59080 | CTTTTGCAGATGGCGCTGTT | ACCCGAGCCTAGACCTCTTT |
| MYB15 | AT3G23250 | AAGCGAATCGGAGCTAGCAG | CCGTCGTGGCTTATGAGTGT |
| CYP81F2 | AT5G57220 | GGGATGAGCCCGAGAAGTTT | CCTAAAGTCGCACCAGGACA |
| HMT | AT5G52750 | GCCTCCAATGAAAGCTGTGT | CCCGGTTTTGTCATCCATTGT |
| MYB51 | AT1G18570 | TGTTGGAGGAGTCTTGTGTTGA | TCATAGACCGGCGTCACATC |
| BCB | AT5G20230 | GTGCAACTGGAGGTGCTACT | TAGTGGTTCCGCTAGAGCCT |
| ACS6 | AT4G11280 | GCAAGGCACGCTGAGATAAC | ATCACACGCCATAGTTCGGT |
| TMP | AT4G28460 | TGTGGTTGTGATGGTGTCGT | CCACCGTCATAGTCGCACTT |
| CRK11 | AT4G23190 | GAAAAACAGCGGCGTCTACC | CGCGATATGGATGCACCTAGT |
| HSP70 | AT3G12580 | TGGCTGAGGCAGATGAGTTC | CCTCCTGCACCACCCATATC |
| TIR | AT1G72900 | GGGGATTTCAGCGGTTTTGG | CGCAGCAGAAATGTCGTCTC |
| HSFA2 | AT2G26150 | TTGGATGTGGGGAGGAAACG | GATTGCTGCAGCGAACAACA |
| PPPDE | AT3G07090 | GGTCGCTCAGTTTTTGGTGG | TTGTGGCTGTGGCCTAAACT |
| Actin2 | AT3G18780 | TCAGATGCCCAGAAGTCTTGTTCC | CCGTACAGATCCTTCCTGATATCC |
