# Supplementary figures and images for: Integrated bioinformatics analysis of As, Au, Cd, Pb and Cu heavy metal responsive marker genes through Arabidopsis thaliana GEO datasets
Source: PeerJ. 2019 Mar 18;7:e6495. doi: 10.7717/peerj.6495 (PMC6428040; doi:10.7717/peerj.6495)

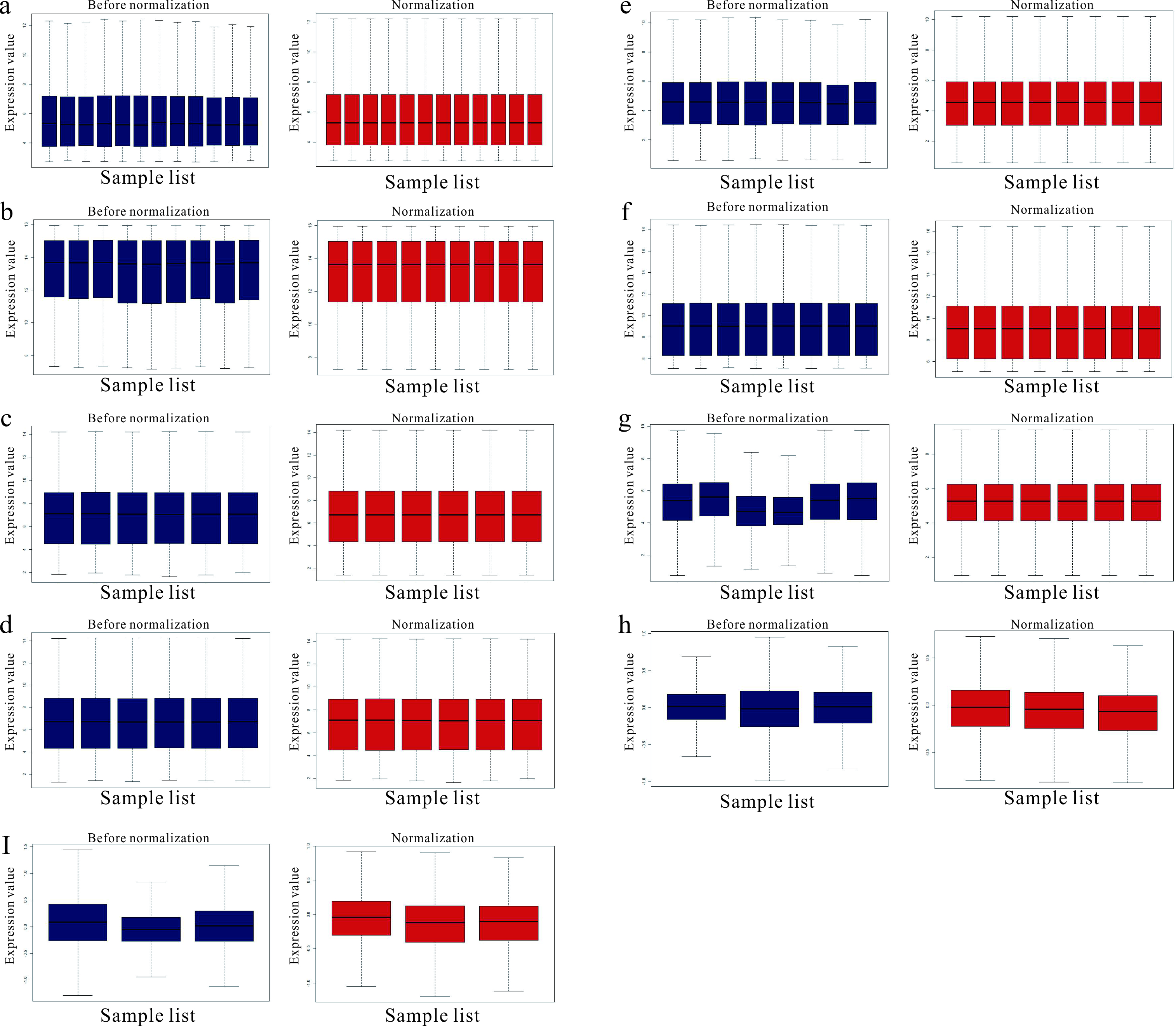

Supplement: Figure S1 — (A) standardization of GSE49037 data of 198; (B)The standardization of GSE49037 data of GPL137; (C)The standardization of GSE55436 data of GPL174; (D)The standardization of GSE55436 data of GPL189; (E)The standardization of GSE94314 data; (F) The standardization of GSE90701 data; (G) The standardization of GSE22114 data; (H) The standardization of 13114 data of GPL177; (I) The standardization of 13114 data of GPL178. The blue bar represents the data before normalization, and the red bar represents the normalized data. [file peerj-07-6495-s009.jpg]

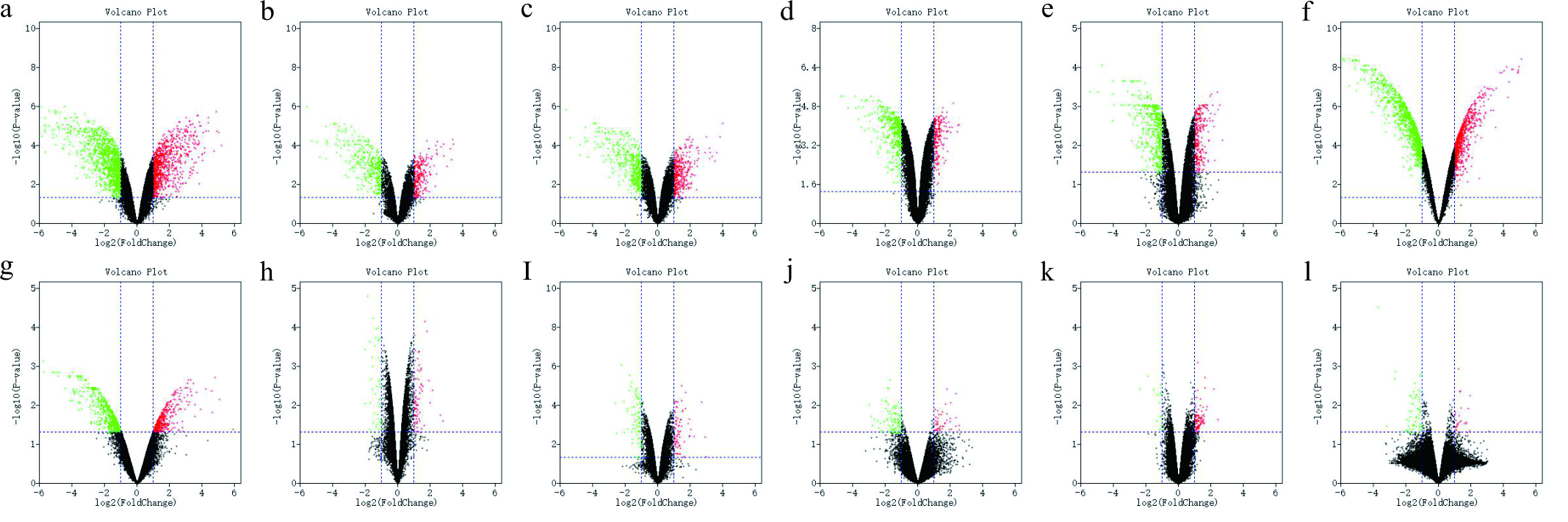

Supplement: Figure S2 — (A) The volcano plot of GSE49037 data of 198; (B)The volcano plot of GSE49037 data of GPL137-1; (C)The volcano plot of GSE49037 data of GPL137-2; (D)The volcano plot of GSE55436 data of GPL174; (E)The volcano plot of GSE55436 data of GPL189; (F)The volcano plot of GSE94314 data of b; (G) The volcano plot of GSE94314 data of c; (H) The volcano plot of GSE90701 data of c; (I) The volcano plot of 90701 data of g; (J) The volcano plot of 22114 data; (K) The volcano plot of 13114 data of a; (L) The volcano plot of 13114 data of b. The red points represent up-regulated genes screened on the basis of |fold change| >2.0, P-value < 0.05. The green points represent down-regulation of the expression of genes screened on the basis of |fold change| >2.0, P-value < 0.05. The black points represent genes with no significant difference. FC is the fold change. [file peerj-07-6495-s010.jpg]

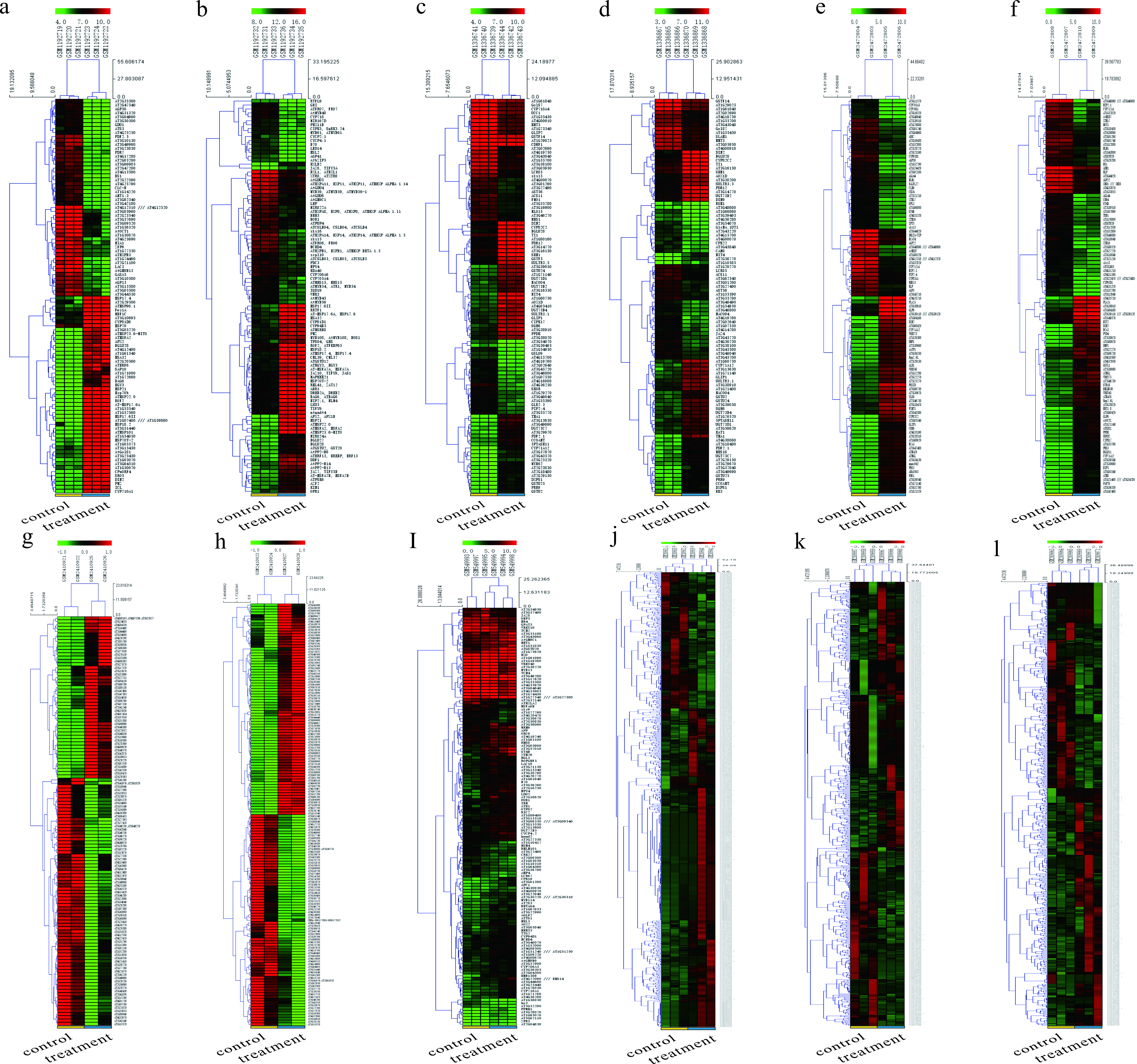

Supplement: Figure S3 — (A) The heatmap of GSE49037 data of GPL198(As treatment); (B) The heatmap of GSE49037 data of GPL137(As treatment); (C) The heatmap of GSE55436 data of GPL174 (Au treatment); (D) The heatmap of GSE55436 data of GPL189 (Au treatment); (E) The heatmap of GSE94314 data of b (Cd treatment); (F) The heatmapeof GSE94314 data of c (Cd treatment); (G) The heatmap of GSE90701 data of c (Cd treatment); (H) The heatmap of GSE90701 data of g (Cd treatment); (I) The heatmap of 22114 data (Cd treatment); (J) The heatmap of 104916 data of c (Cu treatment); (K) The heatmap of 104916 data of s (Cu treatment); (L) The heatmap of 104916 data (Cu treatment). [file peerj-07-6495-s011.jpg]
